# Decoding gene expression dynamics in planktonic and biofilm cells of *Streptococcus mutans*: regulation and role of mutanofactin genes in biofilm formation

**DOI:** 10.3389/froh.2025.1535034

**Published:** 2025-01-17

**Authors:** Muhammad Afzal, Miguel Carda-Diéguez, Susanne Bloch, Leon G. S. Thies, Alex Mira, Christina Schäffer

**Affiliations:** ^1^ Department of Natural Sciences and Sustainable Resources, Institue of Biochemistry, NanoGlycobiology Research Group, Universität für Bodenkultur Wien, Vienna, Austria; ^2^ Department of Genomics and Health, FISABIO Foundation, Valencia, Spain; ^3^ Competence Center for Periodontal Research, University Clinic of Dentistry, Medical University of Vienna, Vienna, Austria

**Keywords:** biofilm, dental caries, MufC regulator, muf operon, mutanofactins, *Streptococcus mutans*, transcriptomics

## Abstract

**Introduction:**

Dental caries is the most prevalent chronic infectious disease globally, with *Streptococcus mutans* recognized as a primary causative agent due to its acidogenicity and robust biofilm-forming ability. In *S. mutans* biofilm formation, the role of autoinducers has been extensively studied, while the influence of other small molecules remains largely unexplored. Mutanofactins, a class of polyketide/non-ribosomal lipopeptide secondary metabolites, are emerging as potential modulators of *S. mutans* biofilm development.

**Methods:**

Transcriptomic analysis was conducted to examine gene expression patterns in *S. mutans* NMT4863 across distinct growth phases and lifestyles, aiming to identify metabolic factors influencing biofilm formation. Transcriptomic profiles were compared between cells in early-, mid-, and late-exponential-, and stationary phase, as well as between planktonic and biofilm cells. Differentially expressed genes were identified, and pathway analyses revealed significant alterations in key metabolic and regulatory pathways. Specifically, the biosynthetic mutanofactin gene cluster was analyzed via quantitative real-time polymerase chain reaction.

**Results:**

Several genes and operons were differentially expressed across the tested growth phases, with 1,095 genes showing differential expression between stationary-phase, planktonic and biofilm cells. Pathway analysis revealed significant changes in ascorbate metabolism, carbohydrate utilization and transport systems, lipoic acid metabolism, bacterial toxin pathways, two-component regulatory systems, and secondary metabolite biosynthesis. Notably, expression of the *muf* gene cluster, was elevated in early exponential-phase cells relative to stationary-phase cells. Additionally, the *mufCDEFGHIJ* genes were identified as components of a single transcriptional unit (*muf* operon). MufC, a transcriptional regulator of the TetR/AcrR-family, acts as a positive regulator of the *muf* operon in strain NMT4863. Bioinformatic analysis pinpointed a 20-bp regulatory sequence in the *muf* operon promoter region (5′-AAATGAGCTATAATTCATTT-3′). Interestingly, the *muf* operon was found to be significantly downregulated in biofilm cells.

**Conclusion:**

This study provides key insights into gene expression dynamics that drive biofilm formation in *S. mutans* NMT4863, with a particular emphasis on the role of the *muf* operon. This operon is governed by the TetR/AcrR-family regulator MufC and plays a central role in biofilm development, offering a novel perspective on the molecular basis of *S. mutans* biofilm formation and resilience.

## 1 Introduction

Dental caries remains a major global health concern, driven by the demineralization of tooth enamel due to acid production within polymicrobial biofilms in the oral cavity (1, 2). This biofilm-mediated disease is exacerbated by poor oral hygiene, which increases biofilm mass and intensifies pH drops after meals. *Streptococcus mutans*, a primary colonizer in the oral microbial ecosystem, plays a pivotal role in biofilm formation and is central to caries pathogenesis (3). Its transition from planktonic cells to robust biofilm communities is tightly linked to its virulence and adaptive strategies, making biofilm regulation a key target for preventive and therapeutic measures (4).

During initial biofilm formation, *S. mutans* adheres to tooth surfaces via surface adhesins and glucan production, forming a protective biofilm matrix (5, 6). Environmental factors such as pH fluctuations, nutrient availability, and antimicrobial agents influence biofilm development and virulence gene expression (3, 7). As the biofilm matures, microbial cells exhibit significant metabolic and gene expression changes, enhancing their resistance to environmental stresses, sustaining acid production, and facilitating enamel demineralization (8, 9). Quorum sensing, a density-dependent signaling mechanism, further regulates the production of extracellular polysaccharides and other virulence factors critical for biofilm stability and pathogenicity (6, 10).

Secondary metabolites, particularly small molecules, are increasingly recognized as critical modulators of microbial biofilm development across diverse ecosystems (11). Polyketides (PKs) and non-ribosomal peptides (NRPs) have garnered significant attention for their multifaceted roles in biofilm dynamics, influencing biofilm architecture, microbial competition, interspecies and inter-kingdom interactions, and immune evasion (12, 13). In *S. mutans*, secondary metabolites, synthesized by PK synthases and NRP synthetases include a range of structurally diverse compounds such as mutanobactins, bacteriocins (13, 14), lantibiotics and microcins (12, 13). These compounds collectively contribute to maintaining ecological balance within the oral microbiota and play pivotal roles in biofilm modulation. Recent studies of *S. mutans* strains with robust biofilm-forming capabilities have identified multiple biosynthetic gene clusters (BGCs) encoding hybrid NRP/PK-synthase-dependent secondary metabolites (15). Among these, BGC1, comprising the *mufA-J* genes including ABC transporter genes, is responsible for the production and cellular export of mutanofactins (MFs)—structurally unique lipopeptides that enhance adhesion and modulate biofilm formation through a distinct physicochemical mechanism (15). Five variants of MFs were detected in the culture extracts of *S. mutans* NMT4863—MF-458, MF-541, MF-539, MF-607 and MF-697; among these, MF-697 is the predominant product, while MF-607 was identified as its immediate biosynthetic precursor (15). Although BGC2 is co-expressed under biofilm-forming conditions, its role in biofilm development appears limited, as its disruption did not impact biofilm structure or stability (15). BGC2 is predicted to comprise 18 genes encoding a diverse array of proteins, including NRPS and PKS enzymes involved in biosynthesis, a transcriptional regulator potentially responsible for gene cluster regulation, modifying enzymes such as FabG and FabZ, and several transport-related proteins. This gene cluster is hypothesized to produce a hybrid polyketide-peptide metabolite, the function of which remains unknown.

Although current evidence underscores the pivotal role of MFs in *S. mutans* biofilm formation (15), their production across distinct bacterial growth phases and growth formats, as well as the underlying gene regulation remain unexplored. This study addresses these gaps, establishing a critical connection between MF biosynthesis and biofilm development in *S. mutans* while identifying a potential regulatory mechanism governing the expression of the *muf* gene cluster (BGC1). The *muf* gene cluster (*mufABCDEFGHIJ*) is organized into two transcriptional orientations: *mufAB* and *mufCDEFGHIJ*. The genes in the latter group (*mufCDEFGHIJ*) are transcribed as a single operon and are regulated by the MufC transcription factor. Through an integrative approach combining whole-transcriptome analysis and targeted genetic manipulations, we delineate the genetic framework underpinning biofilm formation in *S. mutans* NMT4863. Our findings reveal comprehensive gene expression profiles across different growth phases and during biofilm development. Notably, we identify MufC as a transcriptional activator of the *muf* gene cluster, underscoring its central role in regulating mutanofactin production. Additionally, we uncover a conserved 20-bp regulatory motif within the *mufC* promoter region, suggesting its involvement in fine-tuning the expression of this essential activator. These insights advance our understanding of the molecular mechanisms driving biofilm formation and highlight novel targets for therapeutic intervention against *S. mutans*-mediated oral pathologies.

## 2 Materials and methods

### 2.1 Bacterial strain and cultivation conditions

For the planktonic growth of *S. mutans* NMT4863 wild-type (WT), a glycerol stock of the bacterium was streaked onto a Brain Heart Infusion (BHI; containing 2 g/L glucose) plate and incubated overnight at 37°C in 5% CO_2_ (15). A single colony was then inoculated into 5 ml of liquid BHI medium in a test tube and grown overnight at 37°C in 5% CO_2_. Subsequently, the overnight culture was inoculated into fresh BHI medium at a 1:50 ratio (vol/vol) and grown until reaching the early-exponential (equaling OD_600_ ∼0.2), mid-exponential (equaling OD_600_ ∼0.6), late-exponential (equaling OD_600_ ∼0.9), and stationary growth phase (equaling OD_600_ ∼1.2), respectively.

For biofilm growth, bacterial cells from the mid-exponential phase were inoculated into semi-defined biofilm medium (BM) (16) at a dilution of 1:40 (vol/vol). BM contained 58 mM K_2_HPO_4_, 15 mM KH_2_PO_4_, 10 mM (NH_4_)_2_SO_4_, 35 mM NaCl, 20 mM D-glucose, 0.2% casamino acids, 100 µM MnCl_2_ x 4H_2_O and was set to pH 7.4 with HCl before addition of amino acids (1 mM L-arginine-HCl, 1.3 mM L-cysteine-HCl, 4 mM L-glutamic acid, 100 µM L-tryptophan), vitamins (0.05 µM biotin, 10 µM calcium pantothenate, 40 µM niacin, 100 µM pyridoxine-HCl, 1 µM riboflavin, 0.3 µM thiamine-HCl), and 2 mM MgSO_4_ x 7H_2_O. All supplements were filter-sterilized. The bacterial suspension was aliquoted (1 ml) into sterile, clear, tissue culture-treated, flat-bottomed 24-well microtiter plates (Starlab CytoneOne CC7682-7524). The plates were covered and incubated statically at 37°C in 5% CO_2_ for 24 h. Following incubation, the biofilms were washed once with 1 ml of phosphate buffer saline (PBS) to remove planktonic cells and then collected in 1 ml of PBS. The bacterial cells were centrifuged at 4,000 rpm for 10 min at 4°C, and the supernatant was carefully removed. The bacterial cell pellets were stored at −80°C until further use. Additionally, the total biofilm mass was determined. After washing, the biofilms were heat-fixed for 1 h at 60°C (17) and then stained with 500 µl 0.1% crystal violet (CV) for 15 min. To remove excess CV, the wells were washed twice with 1 ml of Milli-Q water, and the air-dried biofilms were photographed using a light pad. For quantification, the CV was mobilized in 1 ml of 30% acetic acid per well (18) and thoroughly mixed before transferring 200 µl into a fresh 96-well plate (655101, Greiner). The absorption was measured using a Tecan Infinite F200 plate reader at 595 nm.

### 2.2 Manipulations at the mutanofactin biosynthesis gene cluster of *S. mutans* NMT4863

For PCR amplifications, chromosomal DNA of *S. mutans* NMT4863 (GenBank accession number AHRZ00000000) (15) was used as a template. All primers (Thermo Fisher Scientific) used in this study are listed in Supplementary Table S1.

#### 2.2.1 Analysis of *muf* gene transcription

**Figure 1:**
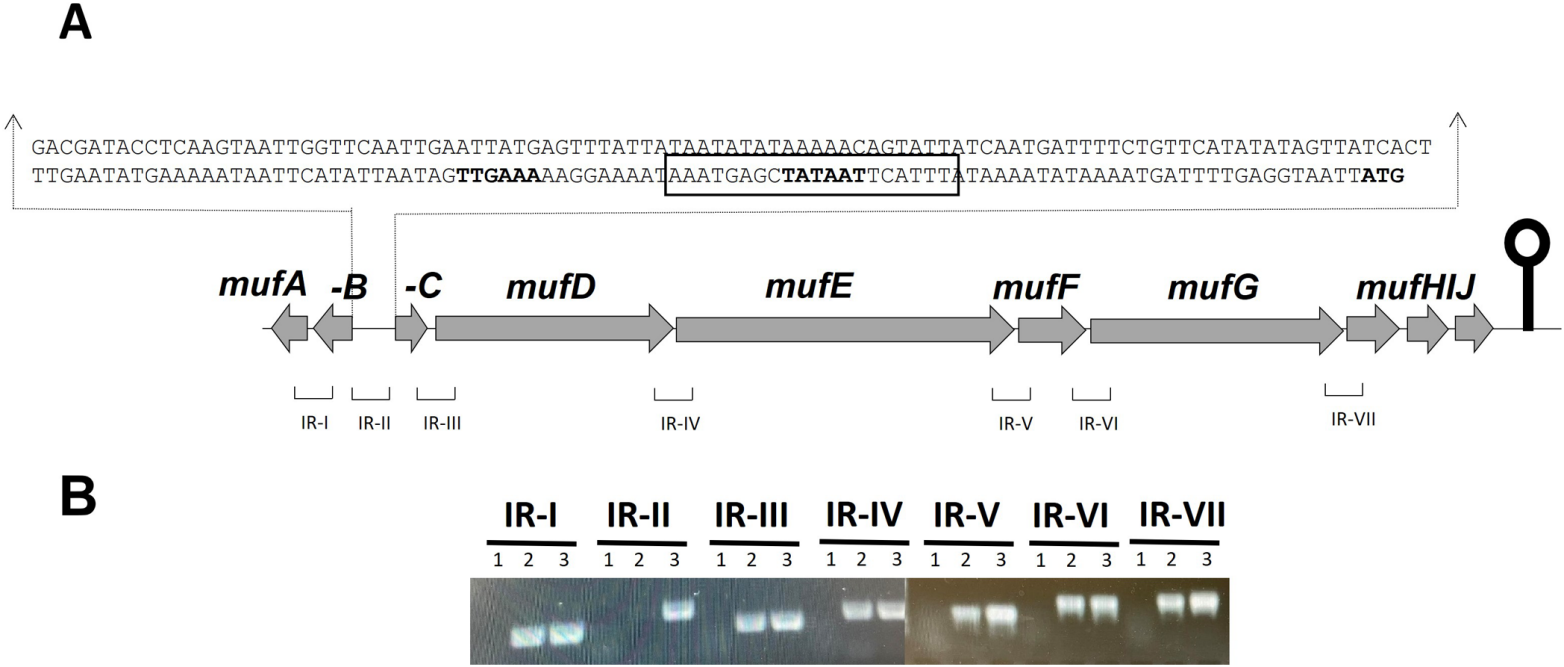
**(A)** Organization of the *muf* operon in *S. mutans* NMT4863. A lollipop structure represents a putative transcriptional terminator, while black arrows indicate the promoter region. Predicted functions of genes are as follows: *mufA*, 1-phosphopantetheinyl transferase; *mufB*, type II thioesterase; *mufC,* AcrR-family transcriptional regulator; *mufD*, surfactin synthase; *mufE*, amino acid adenylation domain containing protein; *mufF*, malonyl∼CoA-acyl carrier protein transacylase; *mufG*, KR domain protein; *mufHIJ,* ABC transporter. **(B)** RT-PCR analysis to conﬁrm the polycistronic nature of the *muf* operon in *S. mutans* NMT4863. RT-PCR was performed on total RNA isolated from NMT4863 WT grown in BHI medium without (1) and with (2) reverse transcriptase treatment using the intergenic region primer pairs. DNA was used as a positive control (3). The size of the RT-PCR products ranges from 100 to 300 bp.

The predicted *muf* gene cluster in *S. mutans* NMT4863 consists of ten genes (Figure 1A) encoding a predicted 1-phosphopantetheinyl transferase (*mufA*), a type II thioesterase (*mufB*), an AcrR-family transcriptional regulator (*mufC*), a surfactin synthase (*mufD*), an amino acid adenylation domain containing protein (*mufE*), a malonyl CoA-acyl carrier protein transacylase (*mufF*), a KR domain protein (*mufG*), and an ABC transporter (*mufHIJ*) (15). To determine whether the putative *muf* gene cluster is transcribed as a single transcriptional unit, we performed RT-PCR on all possible intergenic regions between *mufA* and *mufH* within in the *muf* gene cluster (Figure 1A).

To identify a putative promoter region of *mufC*, we used Genome2D tool (19) and a MEME motif sampler search (20).

#### 2.2.2 Construction of a *mufC*-deficient mutant

To test, whether MufC functions as a transcriptional regulator of the *muf* gene cluster in *S. mutans* NMT4863, a *ΔmufC* (*SMU57_04420*) deletion mutant (MA01-MF) was constructed by allelic replacement with a kanamycin-resistance cassette.

Briefly, primers mufC-1/mufC-2 and mufC-3/mufC-4 were used to generate ∼1-kbp PCR fragments of the up- and down-stream flanking region of *mufC*, incorporating an *AscI* and *NotI* restriction site, respectively. The kanamycin-resistance marker (aminoglycoside phosphotransferase, *kan*; 816 bp) was amplified using primers KanR/KanF) from the pET28a vector (Novagen), with *AscI* and *NotI* restriction sites attached to its ends. The up- and downstream flanking regions of *mufC* were then fused to the kanamycin-resistance gene by restriction and ligation. The resulting ligation products were transformed into *S. mutans* NMT4863 WT through natural transformation using the SigX-inducing peptide (XIP) (21). For this purpose, cells were grown at 37°C until an OD_600_ of ∼0.1 was reached. One ml of the culture was transferred to a 1.5-ml tube, and XIP (sequence: GLDWWSL) was added to a final concentration of 1 µM (21). Cells were incubated at 37°C for 10 min. The ligation mixture was then added to the incubated cells, and the cells were allowed to grow for 3 h at 37°C. Subsequently, the culture was centrifuged for 1 min at 7,000 rpm, and 900 µl of the supernatant was discarded. The cell pellet was resuspended in the remaining medium (∼100 µl) and plated on BHI agar plates supplemented with kanamycin at a concentration of 300 μg/ml. The *S. mutans* NMT4863 *ΔmufC* mutant was confirmed by colony PCR (using primers mufC-seq-1 and mufC-seq-2) and DNA sequencing (Microsynth).

### 2.3 RNA extraction, sequencing and analysis

For RNA-seq analysis, *S. mutans* NMT4863 was grown in liquid BHI medium as described above (15)_._ Cells were harvested at various growth phases, including early-exponential, mid-exponential, stationary phase, and biofilm (see above and Supplementary Figure S1). We utilized three independent biological replicates for each condition.

Total RNA was extracted from bacterial cells using the Bacterial RNA kit (innuPREP, IST Innuscreen). The RNA samples were treated with 2 U of RNase-free DNase I (Invitrogen) to remove potential DNA contamination. The raw RNA product was quantified using a NanoDrop Spectrophotometer, and further quantified using Quant-it™ RiboGreen (Thermo Fisher Scientific). Qualitative analysis was performed with the Fragment Analyzer RNA Analysis, HS RNA kit (15NT) (Agilent). Libraries were prepared using the Illumina Stranded Total RNA Prep, ligation with RiboZero Plus (Illumina), following the manufacturer's instructions. Final library quality control was conducted using Pico488 (Lumiprobe) and the dsDNA 915 Reagent Kit (Agilent). At least 5 + 5 M paired-end reads were produced per sample, which passed Illumina's chastity filter. The reads were then demultiplexed with zero index base mismatches and Illumina adapter trimming using Illumina's bcl2fastq software, version 2.20.0.422 (no further refinement or selection). The quality of the fastq formatted reads was assessed with the FastQC software, version 0.11.9.

Comparison of gene expression between the sample groups was performed using DESeq2 (22). *S. mutans* genes with *p*-values <0.05 and log_2_ fold changes >0.5 were defined as differentially expressed genes (DEGs) for each comparison. The ‘plotPCA’ function within the DESeq2 R package was used for performing Principal component analysis (PCA). Volcano plots were generated using Graphpad Prism version 9.0.0 for Windows (GraphPad Software, Boston, Massachusetts USA, https://www.graphpad.com). Kyoto Encyclopedia of Genes and Genomes (KEGG) pathways were generated using KEGG mapper (https://www.genome.jp/kegg/mapper/). Gene Set Enrichment Analysis (GSEA) was used to examine gene sets from metabolic pathways generated and curated in the KEGG database, using the log_2_ ratio of classes option as a metric and selecting the significantly enriched gene sets of each condition, according with the default *p*-value <0.05. Gene pathways were obtained from KEGG automatic classification and the differently expressed pathways were calculated as the DEGs.

RNA-seq data has been submitted to the National Center for Biotechnology Information (NCBI) and can be freely accessed by BioProject number PRJNA1199217.

### 2.4 Reverse transcription (RT)-PCR and purification for quantitative RT-PCR

For quantitative RT-PCR, *S. mutans* NMT4863 WT and *S. mutans* NMT4863 *ΔmufC* were grown in BHI medium. RNA isolation was performed as described above. The RNA samples were treated with 2U of RNase-free DNase I (Invitrogen) to eliminate any DNA contamination (23). Subsequently, cDNA synthesis was performed on RNA using the High-Capacity cDNA Reverse Transcription Kit (Thermo Fisher Scientific). Briefly, 1 µg of RNA was used for PCR per reaction (total volume 20 µl) in PCR tubes. The samples were incubated at 25°C for 10 min, followed by 2 h at 37°C to allow reverse transcription to proceed, as per the manufacturers’ instructions. The reaction mixtures were then incubated at 80°C for 5 min to inactivate the enzyme (23).

For qRT-PCR, 50 ng of cDNA was used per reaction using the primers listed in Supplementary Table S1. qPCR was carried out using the PowerUp™ SYBR™ Green Master Mix for gene expression analysis (Applied Biosystems). qPCR was performed using CFX Opus 96 (Bio-Rad), with a program consisting of initial denaturation at 95°C for 3 min, followed by 40 cycles at 95°C for 10 s and annealing/extension at 55°C for 30 s. The transcription level of specific genes was normalized to *gyrA* transcription, amplified in parallel using gyrA-F and gyrA-R primers. The results were interpreted using the comparative CT method (24). C_t_ values were determined for each gene, and the expression of the target gene was calculated by the 2^−ΔΔCt^ method, where ΔΔC_t_ = (C_t_^target^–C_t_^gyrA^)_sample –_ (C_t_^target^–C_t_^gyrA^)_control_.

### 2.5 Statistical analyses

For biofilms and qRT/PCR, statistical significance was determined using student's *t*-test. Significance is indicated by: *p < 0.05, **p < 0.01, ***p < 0.001.

## 3 Results

### 3.1 The *muf* genes of *S. mutans* NMT4863 constitute an operon

RT-PCR performed on the intergenic regions of the 10-gene *muf* cluster of *S. mutans* NMT4863 (Figure 1A) revealed that eight of these genes, named *mufCDEFGHIJ*, form a single transcriptional unit (Figure 1B), which we henceforth refer to as the “*muf* operon”. Notably, the first gene of the operon, *mufC*, codes for a putative AcrR-family transcriptional regulator. The *mufA* and *mufB* genes encode a predicted 1-phosphopantetheinyl transferase and a type II thioesterase, respectively (15). These genes are co-transcribed in the opposite direction from the other *muf* genes and, therefore, are not part of the *muf* operon.

### 3.2 qRT-PCR of the *muf* genes during different growth phases of *S. mutans* NMT4863

**Figure 2:**
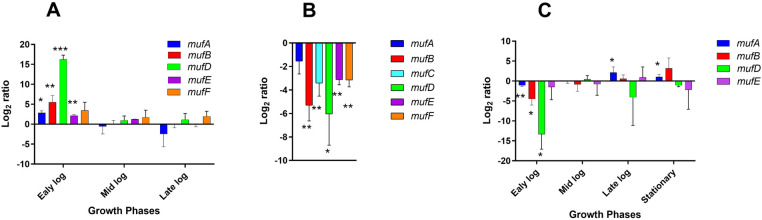
Relative change in expression of the genes belonging to the *muf* operon. **(A)** *S. mutans* NMT4863 WT was grown in BHI medium and harvested at different growth phases. The log_2_ fold-change represents the increase or decrease in the expression of the *muf* genes in the bacterium harvested at the early-exponential, mid-exponential phase, and late-exponential phase compared to that from the stationary phase. **(B)** *S. mutans* NMT4863 WT was grown in BHI medium as planktonic cells and in biofilms. The ratio represents the increase or decrease in the expression of the *muf* genes in the WT during biofilm growth compared to the planktonic stationary phase. **(C)** *S. mutans* NMT4863 WT and *ΔmufC* were grown in BHI and harvested at different growth phases. The log_2_ fold-change represents the decrease or increase in the expression of the *muf* genes in *ΔmufC* compared to the WT. The expression of the *muf* genes was normalized to the housekeeping gene *gyrA*. Results represent the mean and standard deviation of three independent biological replicates (*p < 0.05, **p < 0.01, ***p < 0.001).

To study the expression of the *muf* genes during different growth phases, we harvested *S. mutans* NMT 4863 cells at various stages (early-, mid-, late-exponential and stationary phase) and performed qRT-PCR on the *muf* genes. The results of qRT-PCR show that the expression of the *mufDEF* genes, as well as of *mufAB*, was highest during the early-exponential growth phase when compared to the stationary phase (Figure 2A).

The expression of the *muf* genes in *S. mutans* NMT4863 WT biofilm cells grown overnight (24 h) was compared to planktonic cells from the stationary growth phase. The expression of all tested genes, including *mufCDEF* and *mufAB*, was found to be downregulated during biofilm growth (Figure 2B). This is notable as it may indicate co-regulation of the *muf* operon and the *mufAB* genes.

### 3.3 RNA-Seq analysis of *S. mutans* NMT4863 during different growth phases

**Figure 3:**
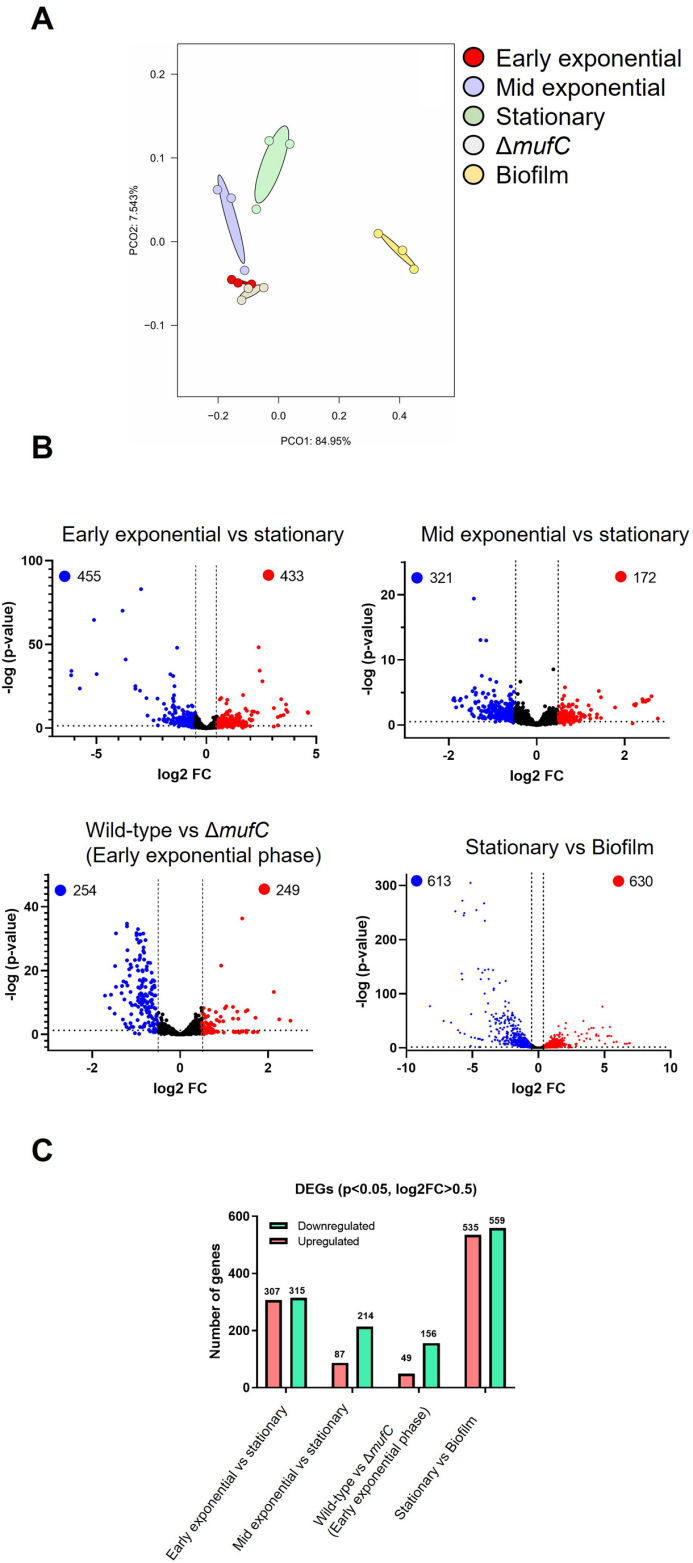
Comparison of transcriptomic profiling of *S. mutans* NMT4863 WT from different growth phases. **(A)** Principal component analysis of the samples. The distance between the groups indicates the similarity of the samples. **(B)** Volcano plot of DEGs. The abscissa indicates the fold change in gene expression, and the ordinate indicates the significance of the gene difference. Red dots represent upregulated genes, blue dots represent downregulated genes, and black dots represent genes that are not significantly different. **(C)** Number of differentially expressed genes in the various growth phases and in biofilm.

Next, we performed RNA-seq analysis to study the global gene expression profile of *S. mutans* NMT4863 WT cells harvested at different growth phases. Principal component analysis (PCA) (Figure 3A) revealed distinctive transcriptomic profiles of planktonic and biofilm cells of *S. mutans*. The PCA results also indicated high levels of correlation and reproducibility within the same sample types (Figure 3A). Volcano plots in Figure 3B illustrate the transcriptomic comparison between different growth phases. Overall, 1,913 *S. mutans* genes were detected via RNA-seq. Among these, 888 genes were differentially expressed (*p*-value <0.05) during the early-exponential phase compared to the stationary phase. Additionally, 493 genes were differentially expressed (*p*-value <0.05) during the mid-exponential phase compared to the stationary phase.

**Figure 4:**
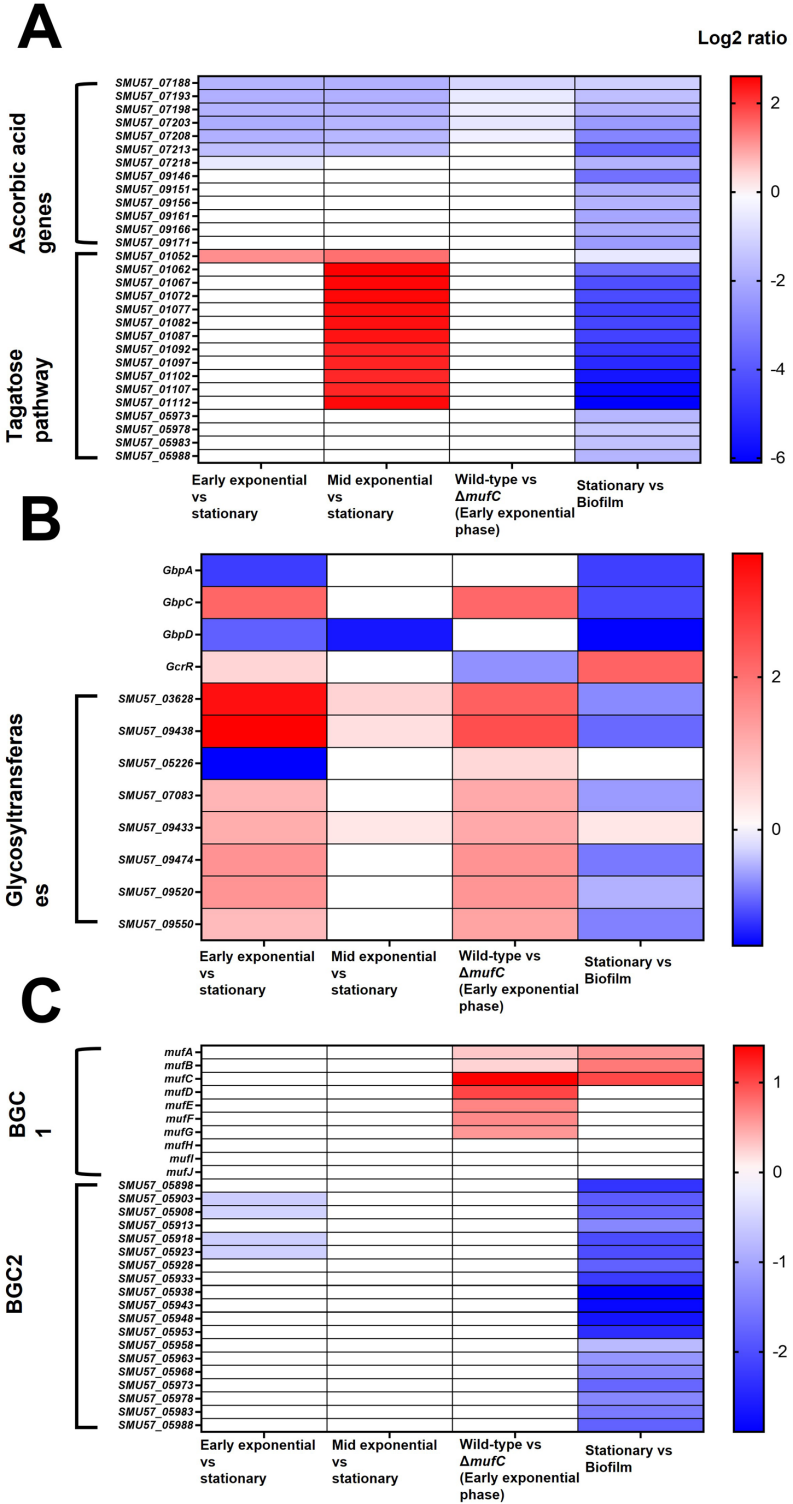
Heatmap of differentially expressed genes (DEGs) across different comparisons. **(A)** Ascorbic acid and tagatose pathway genes. **(B)** Glucan binding proteins (GbPs) and glycosyltransferases (locus tags of *S. mutans* NMT4863). **(C)** Small secondary metabolite biosynthetic gene cluster 1 (BGC1, *muf* gene cluster) and BGC2 (unknown function). Different colors indicate the relative abundance of genes, where red represents higher intensity and blue representing lower intensity.

We further assessed the DEGs (Log_2_ fold change >0.5, *p*-value <0.05) of *S. mutans* NMT4863 WT (Figure 3C). During the early-exponential phase, 307 DEGs were upregulated and 315 were down-regulated compared to the stationary phase. In the mid-exponential phase, 87 DEGs were upregulated and 214 were downregulated compared to the stationary phase. Overall, several *S. mutans* NMT4863 genes involved in carbohydrate transport and metabolism, including those for trehalose, fructose, mannitol and mannose metabolism, were upregulated during the early-exponential phase. In contrast, the tagatose pathway genes involved in the transport and metabolism of lactose and galactose were significantly upregulated during the mid-exponential phase (Figure 4A). Additionally, several putative amino acid transport genes were upregulated during the early-exponential phase. Some *S. mutans* genes relevant to cariogenicity and biofilm formation (25–27) were also significantly upregulated during the early-exponential phase. These genes include several glycosyltransferases and the glucan-binding protein GbpC (Figure 4B). Conversely, the glucan-binding protein GbpA and the putative glucan-binding protein GbpD were significantly downregulated during the early exponential phase.

**Figure 5:**
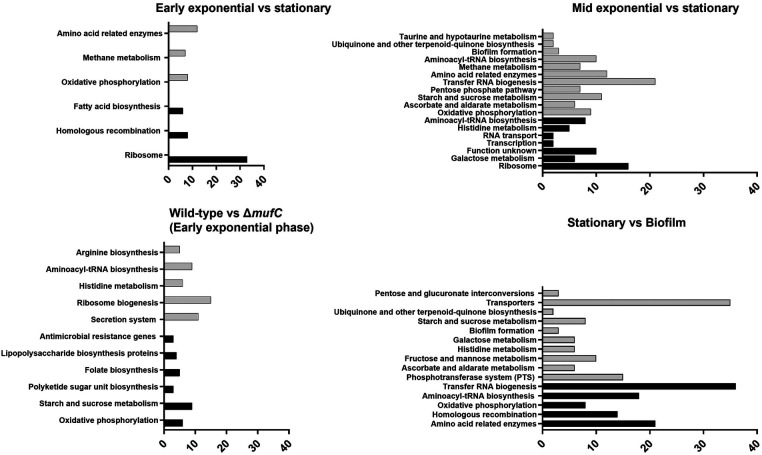
Gene set enrichment analysis (GSEA) of the differentially expressed pathways in *S. mutans* NMT4863 under various planktonic growth phases and in biofilm. The black bars represent upregulated pathways, and gray bars represent downregulated pathways with the number of genes.

Other notable genes that were downregulated during the early-exponential phase compared to the stationary phase included those involved in ascorbic acid metabolism/transport genes, glycogen metabolism, and putative metal (Fe, Zn, Mn, K) transport. GSEA revealed that several KEGG pathways were differentially regulated during the early-exponential phase compared to the stationary phase. Fatty acid biosynthesis genes and the fatty acid biosynthesis pathway were among the upregulated pathways during the early-exponential phase, whereas galactose and histidine pathways were among those upregulated during the mid-exponential phase (Figure 5).

### 3.4 Characterization of the *S. mutans ΔmufC* mutant

#### 3.4.1 qRT-PCR of the mutant

MufC, a putative transcriptional regulator, is encoded by the first gene of the *muf* operon (Figure 1A), suggesting its role in the regulation of this operon. To investigate whether MufC is involved in the regulation of the *muf* operon, we constructed a *mufC* deficient mutant and performed qRT-PCR analysis on *S. mutans* NMT4863 *ΔmufC* compared to the NMT4863 WT grown in BHI and harvested at different growth phases. Figure 2C presents the gene expression analysis results, showing that the *mufAB* and *mufDEF* genes were downregulated in the *ΔmufC* mutant. The downregulation of the *muf* genes was more pronounced during the early-exponential phase, aligning with our gene expression results that indicate that the *muf* operon is upregulated during this growth phase (Figure 2A). These findings suggest that MufC functions as a positive transcriptional regulator of the *muf* operon.

#### 3.4.2 RNA-Seq analysis of the mutant

To further validate our qRT-PCR results and examine the impact of *mufC* deletion on the transcriptome of *S. mutans* NMT4863 WT, we conducted RNA-seq analysis. We compared the transcriptome of NMT4863 *ΔmufC* with that of the NMT4863 WT (Figure 2), both grown in BHI and harvested at the early-exponential phase. This growth phase was selected for the RNA-seq analysis, because our qRT-PCR results indicated significant downregulation of the *muf* genes during the early-exponential phase in the *ΔmufC* deletion mutant. A total of 503 genes were differentially expressed (*p*-value <0.05) in this comparison. Specifically, 156 differentially expressed genes (DEGs) were downregulated, and 49 genes were upregulated in the NMT4863 WT compared to *ΔmufC* (Log_2_ fold change >0.5, *p*-value <0.05). The *muf* operon was also significantly downregulated in the *ΔmufC* mutant. These findings further confirm that MufC activates the expression of the *muf* operon, and this activation is lost in the absence of *mufC*.

Several other genes were significantly downregulated in *ΔmufC*, including those encoding glycosyltransferases (*SMU57_03628*, *SMU57_05226*, *SMU57_07083*, *SMU57_09433*, *SMU57_09438*, *SMU57_09474*, *SMU57_09520* and *SMU57_09550*) and the glucan-binding protein GbpC (Figure 4B). Additionally, several genes were upregulated in *ΔmufC*, including competence genes (*comGF*, *comYA*, *SMU57_06613*, *comYC*, *SMU57_01517*). Gene set enrichment analysis (GSEA) revealed that several KEGG pathways were differentially regulated in *ΔmufC* (Figure 5). Amino acid pathways were upregulated in the *ΔmufC* mutant, whereas antimicrobial resistance, lipopolysaccharide biosynthesis, folate biosynthesis, polyketide sugar unit, sucrose/starch metabolism, and oxidative phosphorylation pathways were significantly downregulated in *ΔmufC* (Figure 5).

#### 3.4.3 Biofilm formation of the mutant

**Figure 6:**
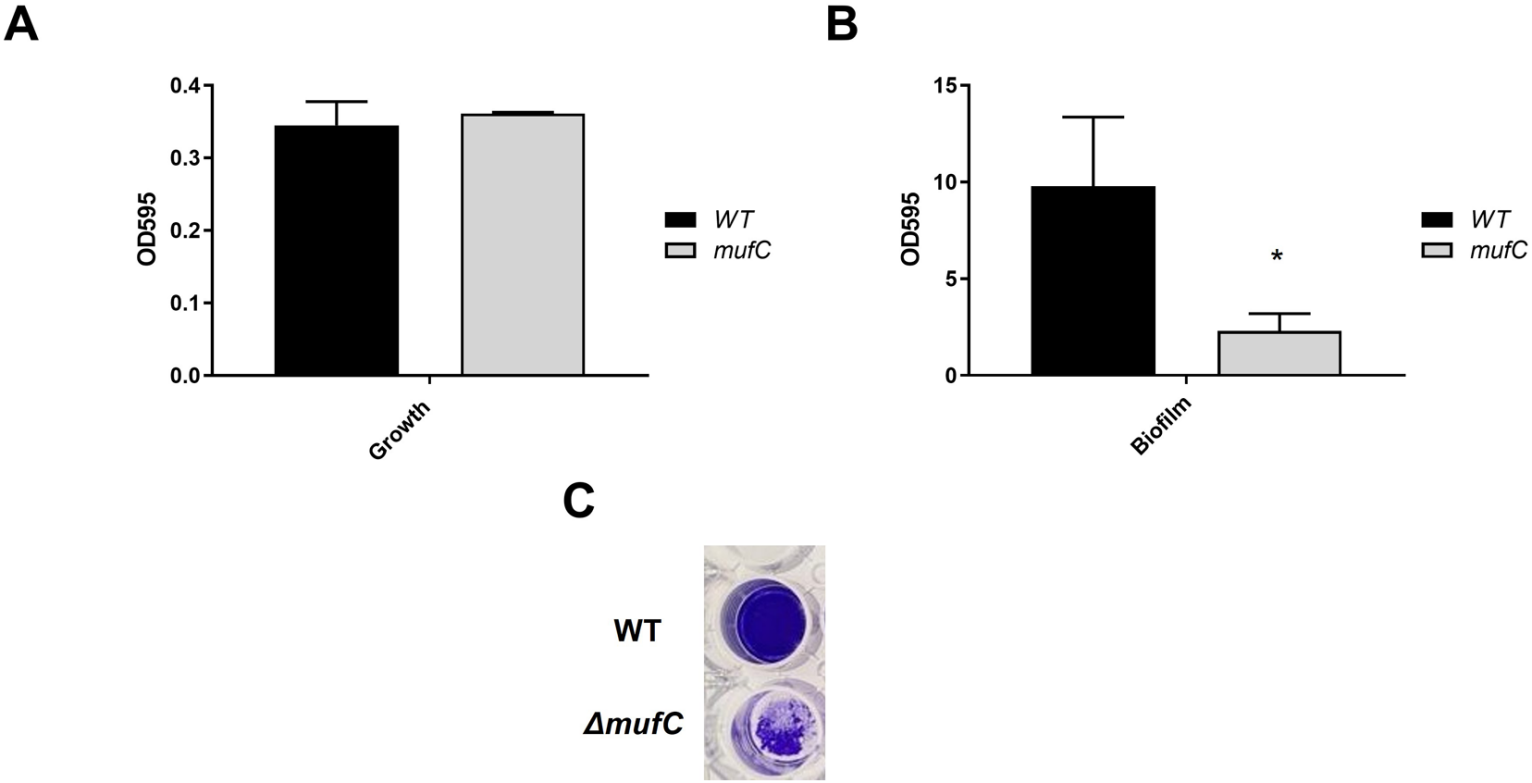
Quantitative analysis of planktonic growth **(A)** and biofilm formation **(B)** of *S. mutans* NMT4863 WT and *ΔmufC* grown in BM medium in 24-well polystyrene plates for 24 h. **(C)** Image of typical biofilms formed by *S. mutans* NMT4863 WT and *ΔmufC* in the assays upon crystal violet staining. The data represents mean ± standard deviation (*n* ≥ 3, *p < 0.05).

To assess the impact of *mufC*, and consequently the *muf* operon, on the biofilm formation of *S. mutans* NMT4863, we compared the total biofilm mass of the mutant to that of the wild-type bacterium after 24 h of biofilm growth. The CV-assay revealed a significant decrease of biofilm in the mutant (Figure 6), confirming the results of the *muf* gene expression analysis (Figure 2C).

### 3.5 *Muf* operon promoter analysis and alignment with putative operator site

**Figure 7:**
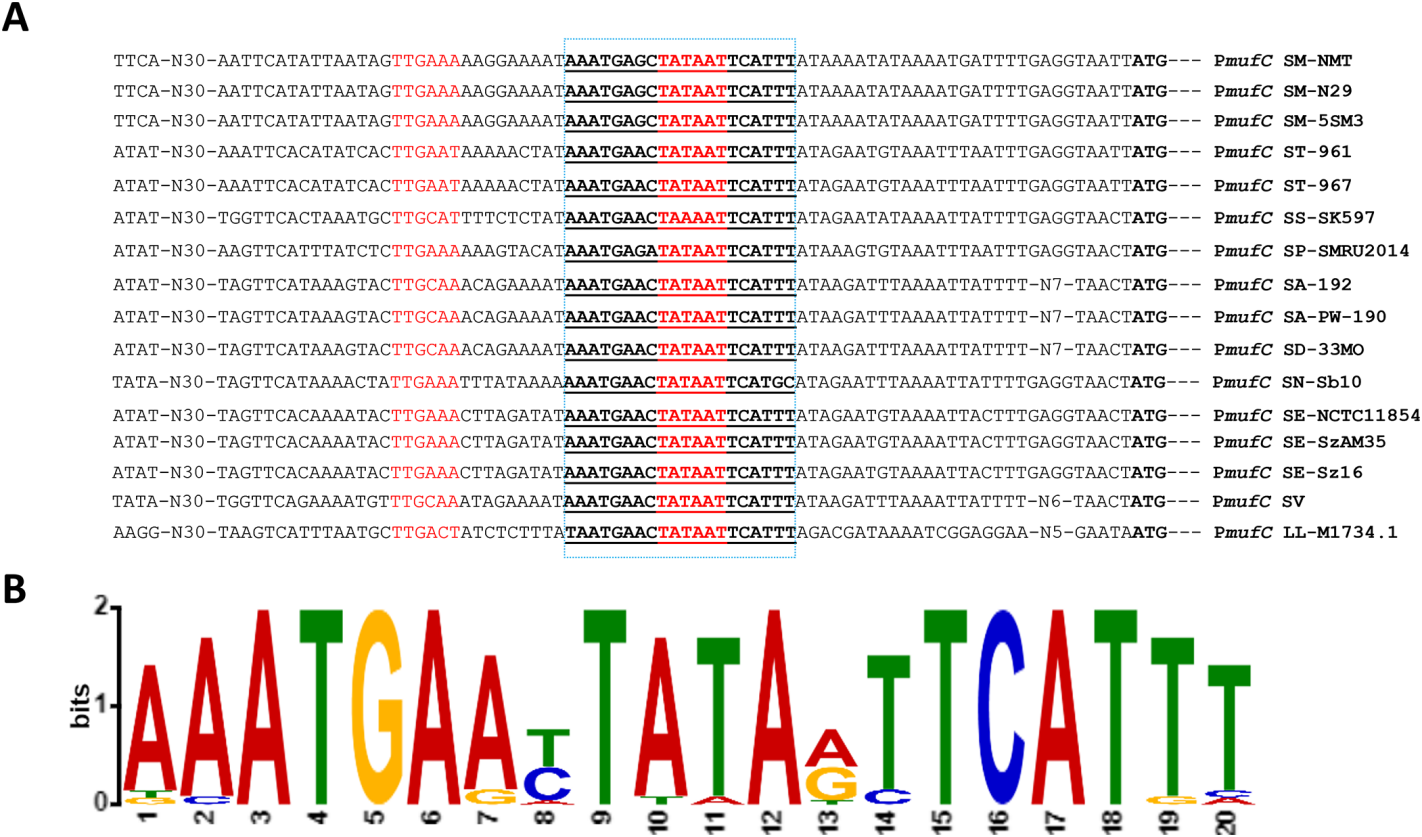
Identification of the *mufC* operator site in the *mufC* promoter (p*mufC*). **(A)** Position of the MufC operator site in the *mufC* promoter of different streptococci. Translational start sites are italicized, and putative MufC operator sites are in boldface and underlined. **(B)** Weight matrix of the identified MufC operator site in the p*mufC* of different streptococci. SM, *S. mutans*; ST, *S. thermophilus*; SS, *S. mitis*; SP, *S. pneumoniae*; SA, *S. agalactiae*; SD, *S. macedonicus*; SN, *S. equinus*; SE, *S. equi* subsp. *zooepidemicus*; SV, *S. vestibularis*; LL, *Lactococcus lactis*.

MufC, a putative TetR/AcrR-family transcriptional regulator, is the first gene of the *muf* operon in *S. mutans* NMT4863. Using the Genome2D tool (19) and a MEME motif sampler search (20), we identified a 20-bp palindromic sequence upstream of *mufC* in *S. mutans* NMT4863 WT -5′-AAATGAGCTATAATTCATTT-3′ (Figure 7A)- which may serve as the regulatory site for *mufC* (P*mufC*) in *S. mutans* NMT4863.

To investigate whether this putative *mufC* regulatory site is conserved across different streptococci, we aligned the upstream regions of *mufC* from various species - *S. mutans* (GenBank accession numbers AHRZ00000000, AHRY00000000 and AHRU00000000)*, Streptococcus thermophilus* (GenBank accession numbers LR822026.1 and LR822025.1), *Streptococcus mitis* (GenBank accession number NZ_AEDV00000000.1), *Streptococcus pneumoniae* (GenBank accession number NZ_CKYA00000000.1)*, Streptococcus agalactiae* (GenBank accession numbers NZ_MAWT00000000.1 and NZ_WNIT00000000.1), *Streptococcus macedonicus* (GenBank accession number NZ_JNCV00000000.1), *Streptococcus equinus* (GenBank accession number NZ_FNJY00000000.1), *Streptococcus equi* subsp. *zooepidemicus* (GenBank accession number LR590471.1, NZ_JATY00000000.1 and NZ_JATW00000000.1), *Streptococcus vestibularis* (GenBank accession number NZ_CABHNJ000000000.1), and *Lactococcus lactis* (GenBank accession number NZ_VJWU00000000.1). The analysis suggests that the *mufC* promoter sequence is highly conserved in all streptococci included in the comparison (Figure 7B).

### 3.6 RNA-Seq analysis of *S. mutans* in biofilm

The PCA revealed distinctive transcriptomic profiles of *S. mutans* biofilm cells compared to those of planktonic cells (Figure 3A). PCA results also demonstrate significant correlation and consistency within the same sample types, both planktonic and biofilms. Volcano plots in Figure 3B illustrate the transcriptomic comparison between *S. mutans* planktonic cells and biofilms. A total of 1,243 genes were differentially expressed (*p*-value <0.05) in this comparison. Specifically, 535 DEGs were upregulated and 559 were downregulated in planktonic cells compared to the biofilm condition (Log_2_ fold change >0.5, *p*-value <0.05).

Overall, several *S. mutans* genes involved in carbohydrate transport and metabolism, including those for trehalose, sorbitol, fructose, maltose, mannitol and mannose, were upregulated during biofilm growth. The tagatose pathway genes involved in the transport and metabolism of lactose and galactose were also significantly upregulated in the biofilm (Figure 4A). Additionally, several *S. mutans* genes associated with caries and biofilm formation were significantly upregulated during biofilm growth. These include several glycosyltransferases (SMU57_03628, SMU57_05226, SMU57_07083, SMU57_09433, SMU57_09438, SMU57_09474, SMU57_09520 and SMU57_09550), putative glucan-binding protein GbpA, and the glucan-binding proteins GbpC and GbpD. A gene cluster, referred to as BGC2 by Li et al. (15), was significantly upregulated during biofilm formation (Figure 4C). Other notable genes differentially expressed during biofilm growth included those involved in ascorbic acid metabolism/transport, glycogen biosynthesis and metabolism, and putative metal (Cu, Fe, Zn, Mn, K, Na, Co) transport. Moreover, several putative amino acid genes were downregulated in biofilm. Our GSEA revealed that several KEGG pathways were differentially regulated in biofilm (Figure 5). Carbohydrate pathways were among the most upregulated during biofilm growth, whereas amino acid pathways were downregulated in the biofilm (Figure 5).

## 4 Discussion

*S. mutans* produces various secondary metabolites, including the recently discovered MFs, which are implicated in biofilm formation and pathogenicity (15, 28, 29). While the predicted *muf* gene cluster responsible for MF production has been sequenced and enzyme functions in the hybrid NRP/PKs biosynthesis pathway have been predicted (15), the regulatory mechanism governing this gene cluster across different growth phases and in planktonic versus biofilm state as well its connection to the global gene expression profile of *S. mutans* remain unexplored.

Bacterial NRP-PK synthetase-dependent gene expression is regulated through diverse mechanisms, including specific transcription factors, integration of environmental signals, and coordination by global regulators (30). In *S. mutans*, a TetR-family regulator controls the mutanobactin gene cluster, regulating the production of mutanobactin D. This compound affects the yeast-to-hyphae transition in *Candida albicans* and modulates interspecies interactions within the oral microbiome (31). TreR, another regulator in *S. mutans*, governs mutacin production and stress responses; its deletion disrupts the LytTR system, reduces ROS tolerance, and impairs mutanobactin production, highlighting its role in oxidative stress and pathogenicity (32). The VicRK two-component system also regulates bacteriocin production, biofilm formation, and cell viability by modulating the competence-stimulating peptide (CSP) and interacting with the ComDE system. VicRK-deficient mutants exhibit increased autolysis and extracellular DNA release, underscoring VicRK's role in stress tolerance and pathogenicity (33).

**Figure 8:**
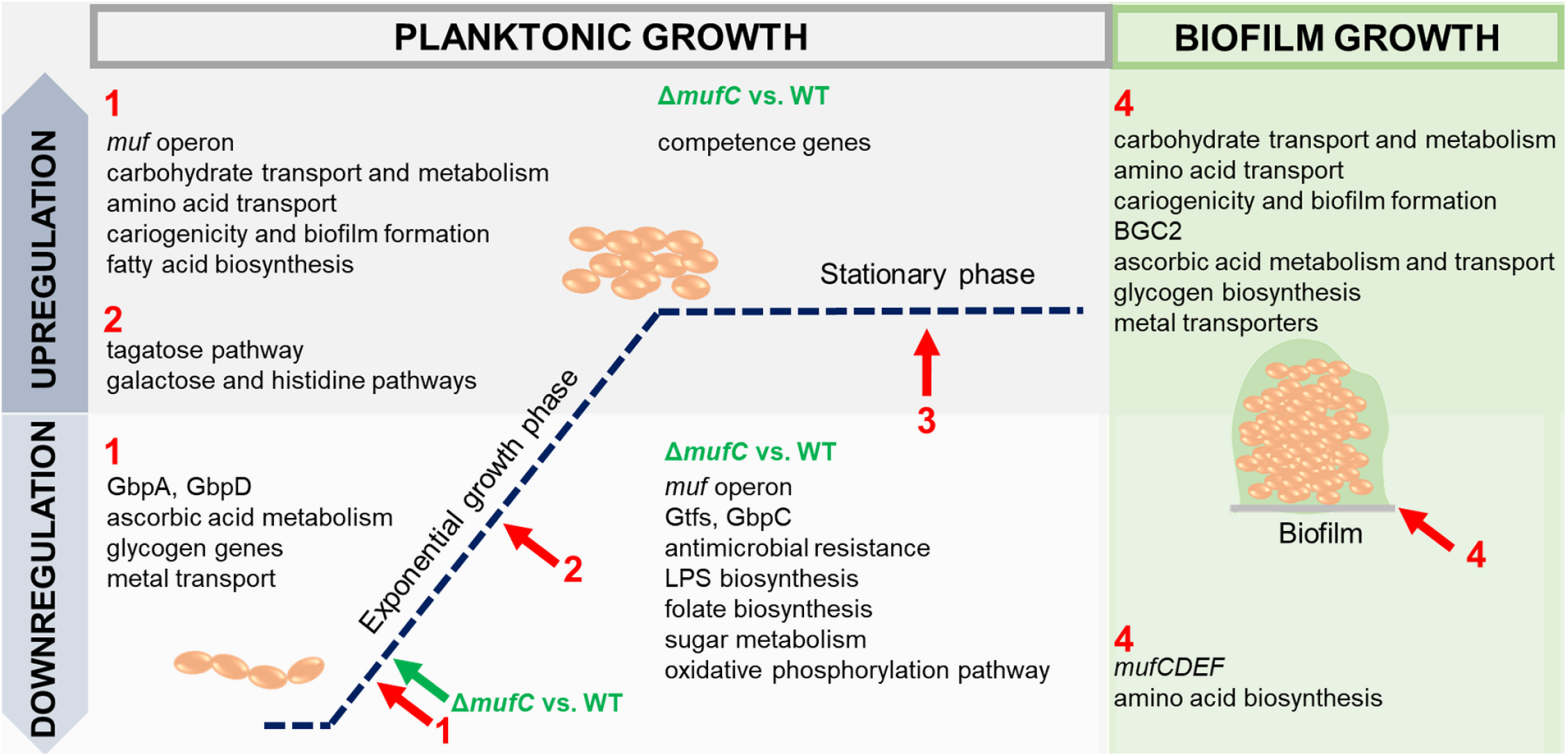
Overview of up-and downregulated genes in *S. mutans* NMT4863 WT and the *ΔmufC* mutant in different phases of planktonic growth compared to the stationary phase and in biofilm (1, early-exponential phase; 2, mid-exponential phase; 3, stationary phase; and 4, biofilm). Arrows represent the time points of cell harvesting.

This study explores the role of MFs in biofilm formation by *S. mutans* NMT4863, with a focus on the regulation of the *muf* operon (Figure 8). Although *mufD*, *mufE*, and *mufF* belong to the same operon, their expression levels and patterns differ. This phenomenon is commonly observed in bacterial operons and may result from post-transcriptional regulatory mechanisms, such as differences in mRNA stability, translational efficiency, or the impact of secondary mRNA structure. Moreover, we reveal that MufC, a putative TetR/AcrR family transcriptional regulator, acts as an activator of the *muf* operon. TetR regulators are known for their roles in antibiotic resistance and the regulation of small molecule export (34). It is likely that *mufC* influences gene cluster expression through direct or indirect mechanisms; however, its precise mode of action, interactions with other regulatory networks, and the environmental cues governing its expression remain to be elucidated. These insights enhance our understanding of secondary metabolite regulation in *S. mutans* and its adaptation to diverse growth and stress conditions. Notably, a conserved MufC regulatory site was identified within the *muf* operon promoter, with homologs observed across multiple streptococci, suggesting a similar regulatory function (Figure 7A).

Bacterial NRP production often varies across different growth phases. For instance, *Bacillus subtilis* produces surfactin during the exponential phase, aiding in surface tension reduction and biofilm formation (35, 36). Similarly, *Streptomyces* synthesize diverse NRPs, such as the antibiotic tyrocidine during exponential growth and the antifungal candicidin during the stationary phase (37, 38). Cyanobacteria produce NRPs, such as cyanopeptides, during exponential growth, which play roles in allelopathy and grazer defense (39). In our study, *muf* operon gene expression was notably upregulated during the early-exponential phase compared to the stationary phase, highlighting its potential role in biofilm initiation, such as surface attachment and extracellular matrix production. This aligns with the critical need for bacteria to establish biofilms during early growth (40). Further research into MF production across growth phases, including the synthesis of MFs to obtain pure lipopeptides for targeted activity assays, is necessary to validate these findings. Testing under varied conditions and across species will provide deeper insights into MF functionality and its broader applications.

*S. mutans* employs a diverse array of genes and pathways to establish biofilms, a process involving adhesion, aggregation, and the production of extracellular polymeric substances (EPS) (41). Adhesins and lectins mediate initial attachment to tooth surfaces and intercellular interactions (42). Carbohydrate metabolism pathways are critical for utilizing dietary and host-derived sugars, with transporters and enzymes facilitating nutrient uptake to support growth and biofilm formation (43, 44). These pathways are intricately linked to quorum sensing and regulatory networks, such as AI-2-mediated signaling, which modulates EPS production, stress response, and virulence factors, all vital for biofilm development (45, 46). In biofilm cells, our transcriptomic analysis identified upregulation of several carbohydrate utilization and transport systems, including those for lactose, galactose, sucrose, fructose, and mannitol. Notably, tagatose pathway genes (*lacABCD*) involved in galactose metabolism were significantly upregulated, consistent with findings in dual-species biofilms of *S. mutans* and *C. albicans* (47). Ascorbic acid utilization systems also showed significant upregulation, with genes such as *ptxA* and *ptxB* (L-ascorbate phosphotransferase system) in *S. mutans* UA159 linked to biofilm formation, growth rate, acid tolerance, and EPS production, particularly when L-ascorbate serves as the primary carbon source (48). Notably, a previous study found that an L-ribulose 5-phosphate 4-epimerase involved in ascorbic acid metabolism was significantly upregulated in *S. mutans* biofilms grown on polystyrene vs. hydroxyapatite (49). Glucosyltransferase genes, such as those encoding GtfB, GtfC, and GtfD (50, 51), responsible for synthesizing glucans as a major extracellular matrix component (52), were significantly upregulated during biofilm growth of *S. mutans* NMT4863. This upregulation likely enhances glucan production, promotes adhesion, intercellular aggregation, and biofilm maturation, thereby strengthening biofilm structure and contributing to dental caries development (52–54). Additionally, several stress-related genes, including those for the ATP-dependent protease ClpE, the putative chaperonin GroEL and co-chaperonin GroES, and the heat shock proteins GrpE and DnaK, were upregulated in biofilms, suggesting a role in adapting to environmental stresses. These findings align with previous studies on polystyrene vs. hydroxyapatite biofilms (49) and underscore the importance of stress response mechanisms in robust biofilm formation. This is supported by another study investigating the dynamics of the *S. mutans* transcriptome in response to starch and sucrose, which showed complex remodeling in response to changing carbohydrate sources for biofilm formation (55). In biofilm cells grown with sucrose for 21 h, a duration comparable to that in the present study, several genes related to sugar metabolism were upregulated, such as those involved in maltose/maltotriose uptake and glycogen synthesis, as well as the GroEL/GroES chaperones in sucrose/starch biofilms, indicating that presence of starch hydrolysates may cause environmental stress.

Another biosynthetic gene cluster, BGC2, comprising 19 genes, showed significant upregulation in our biofilm transcriptome analysis. While the product of this cluster remains unidentified, it is hypothesized to be one of seven natural product BGCs in *S. mutans* (15). A study by Li et al. reported that deletion of BGC2 did not significantly impact biofilm formation of *S. mutans* (15). However, the consistent upregulation of all BGC2 genes in our experiments highlights its potential importance and warrants further investigation. Understanding the role and regulation of BGC2 could provide critical insights into the complex mechanisms underlying *S. mutans* biofilm formation.

In conclusion, this study unveils global gene expression profiles of *S. mutans* linked to distinct growth phases and biofilm development, and elucidates a critical regulatory mechanism underlying MF production, emphasizing the pivotal role of MufC as a transcriptional activator within the *muf* operon. Our findings highlight key genetic and metabolic adaptations that enable *S. mutans* to form and maintain biofilms, thereby facilitating its pathogenic role in dental caries. These insights into the molecular regulation of biofilm formation open promising avenues for therapeutic strategies targeting dental caries. The detailed exploration of the regulatory network governing secondary metabolite biosynthesis in *S. mutans* identifies potential targets for intervention, not only in dental caries management but also in addressing broader challenges associated with biofilm-related bacterial pathogenesis.

## Data Availability

RNA-seq data generated in this project has been submitted to the National Center for Biotechnology Information (NCBI) and can be freely accessed by BioProject number PRJNA1199217.
