# Supplementary material for: Decoding gene expression dynamics in planktonic and biofilm cells of Streptococcus mutans: regulation and role of mutanofactin genes in biofilm formation
Source: Front Oral Health. 2025 Jan 17;6:1535034. doi: 10.3389/froh.2025.1535034 (PMC11782227; doi:10.3389/froh.2025.1535034)
Supplement: Supplementary file 1 [file Datasheet1.pdf]

Supplementary Material

Decoding Gene Expression Dynamics in Planktonic and Biofilm Cells of *Streptococcus mutans*: Regulation and Role of Mutanofactin Genes in Biofilm Formation

Muhammad Afzal[1], Miguel Carda-Diéguez[2], Susanne Bloch[1,3], Leon G. S. Thies[1], Alex Mira[2], Christina Schäffer[1,*]

[1] *NanoGlycobiology* Research Group, Institute of Biochemistry, Department of Natural Sciences and Sustainable Resources, Universität für Bodenkultur Wien, Vienna, Austria

[2] Department of Genomics and Health, FISABIO Foundation, Valencia, Spain.

[3] Competence Center for Periodontal Research, University Clinic of Dentistry, Medical University of Vienna, Vienna, Austria

***Correspondence:**
Christina Schäffer, christina.schaeffer@boku.ac.at

Supplementary Table S1: List of primers used in this study.

Name	Nucleotide Sequence (5'→3')	Restriction site
**Construction of Δ*mufC* mutant**		
mufC_KO_1	GAATGGGAAATACTATAGAAGATACC	-
mufC_KO_2	GCTAT<u>GGCGCGCC</u>CATCTCTTGCCATAATTACCTC	*AscI*
mufC_KO_3	CGATT<u>GCGGCCGC</u>GGTTTCCATTGTTGCAGAGATAC	*NotI*
mufC_KO_4	GCGTTTATTTCGCCTGTTCCTG	-
mufC-seq-1	GTTCATATATAGTTATCACTTTGA	-
mufC-seq-2	CCATCTTAACCTCCTTATTCAT	-
KanF	GCTAT<u>GGCGCGCC</u>CAAGGGGTGTTATGAGCCA	*AscI*
KanR	CGATT<u>GCGGCCGC</u>CCGCTCATGAATTAATTCTTAG	*NotI*
**Quantitative RT-PCR primers**		
mufA-q1	CCAAGTTGGATTCTCAAACCA	-
mufA-q2	TAATTGATCTTTGAATATCACTCCC	-
mufB-q1	CATCATCAGTCTACTCATCTTGG	-
mufB-q2	GACCACCTAGACTATGTCCAA	-
mufC-q1	GGCAGGAGTTTCTGTGGGTG	-
mufC-q2	CTTTTCGTAAGACTTCCATTTGC	-
mufD-q1	ACGAAGGTGGCTTTTACAA	-
mufD-q2	TAACTTTCAAACTCTTTCCTGTC	-
mufE-q1	GATCAGCCCAAAAAGTTAGC	-
mufE-q2	GTGATCTCAAGTTTAGACAACG	-
mufF-q1	CCGTATTTCCTGGGCAAGG	-
mufF -q2	CCAATCATATTCATGGCTTTGT	-
gyrA-q1	GAGTGTTATTGTTGCTCGGGC	-
gyrA-q2	GCGGCTTGTCAGGAGTAACC	-
**RT PCR Primers**		
IR-I-1	TAATTGATCTTTGAATATCACTCCC	-
IR-I-2	ACCCTGTTGAAGGAGATCACT	-

IR-II-1	GACCACCTAGACTATGTCCAA	-
IR-II-2	GAGGTAATTATGGCAAGAGATG	-
IR-III-1	GGTTTCCATTGTTGCAGAGATAC	-
IR-III-2	CACCTTCGTTGGATATTCTTGG	-
IR-IV-1	GTCGATAGACATTCTAGATACGG	-
IR-IV-2	GTGATCTCAAGTTTAGACAACG	-
IR-V-1	GTGACGGAGGAACTACGATAAG	-
IR-V-2	GCCCAGGAAATACGGCTGC	-
IR-VI-1	CATATTGGAGGTTTAAAATGGGG	-
IR-VI-2	GGGATCAATTAATCGTGATTCTGC	-
IR-VII-1	CGAAATGAGGTATTCATTGAGTCAG	-
IR-VII-2	CTTGAGGAACAATTCCGAGTTG	-

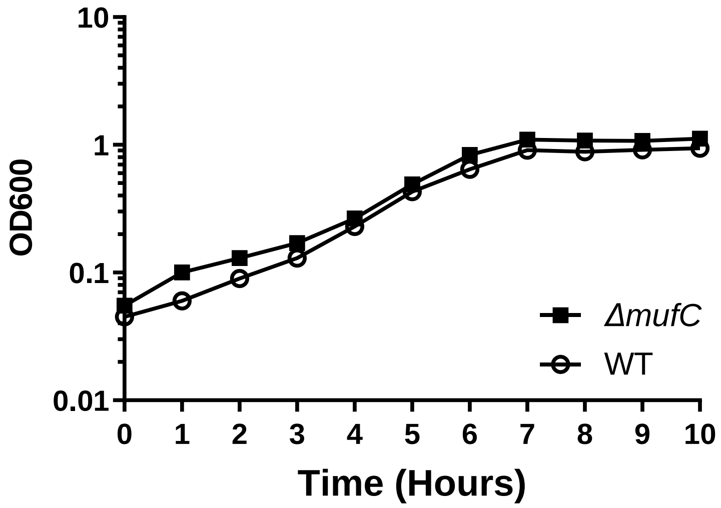

Supplementary Figure S1: Growth curve of *S. mutans* NMT4863 wild-type (WT) and Δ*mufC* in brain heart infusion (BHI) broth.

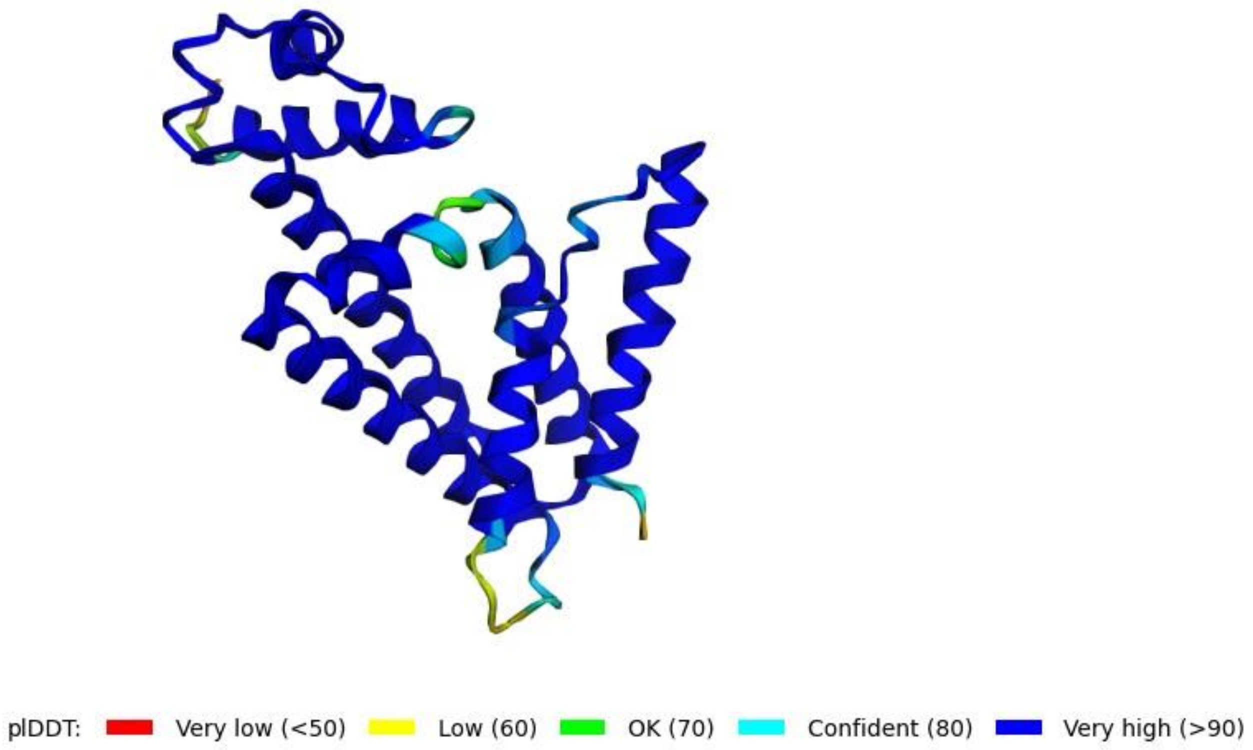

Supplementary Figure S2: Putative structure of MufC colored by pLDDT using the Alphafold palette. AlphaFold2 using MMseqs2 (in ColabFold v1.5.5) was applied with the default settings creating 5 ranked but not relaxed models. All 5 created models have acceptable confidence (ranked 1 model: pLDDT=92.2 pTM=0.858).
